# Evaluation of the Effectiveness of Malaria Vector Control Measures in Urban Settings of Dakar by a Specific *Anopheles* Salivary Biomarker

**DOI:** 10.1371/journal.pone.0066354

**Published:** 2013-06-20

**Authors:** Papa Makhtar Drame, Abdoulaye Diallo, Anne Poinsignon, Olayide Boussari, Stephanie Dos Santos, Vanessa Machault, Richard Lalou, Sylvie Cornelie, Jean-Yves LeHesran, Franck Remoue

**Affiliations:** 1 Institut de Recherche pour le Développement (IRD), UMR-MIVEGEC (IRD224–CNRS5290– Universites Montpellier 1 et 2), Montpellier, France; 2 Institut de Recherche pour le Développement, UMR-216 (Mère et Enfant face aux Infections Tropicales), Faculté des sciences pharmaceutiques, Paris, France; 3 Université d’Abomey-Calavi, International Chair in Mathematical, Physics and Applications (ICMPA-UNESCO Chair), Laboratoire d’ Etude et de Recherche en Statistique Appliquée et Modélisation (LERSAM), Cotonou, Benin; 4 Institut de Recherche pour le Développement, UMR-151, Campus International de Recherche UCAD/IRD, Dakar, Sénégal; 5 Observatoire Midi-Pyrénées, Laboratoire d’Aérologie, Toulouse, France; 6 IRD, UMR-151, Université de Provence, Marseille, France; 7 Institut de Recherche pour le Développement/Centre de Recherche Entomologique de Cotonou, UMR-MIVEGEC (IRD224– CNRS5290– Universities of Montpellier 1 and 2), Cotonou, Benin; Kenya Medical Research Institute (KEMRI), Kenya

## Abstract

Standard entomological methods for evaluating the impact of vector control lack sensitivity in low-malaria-risk areas. The detection of human IgG specific to *Anopheles* gSG6-P1 salivary antigen reflects a direct measure of human–vector contact. This study aimed to assess the effectiveness of a range of vector control measures (VCMs) in urban settings by using this biomarker approach. The study was conducted from October to December 2008 on 2,774 residents of 45 districts of urban Dakar. IgG responses to gSG6-P1 and the use of malaria VCMs highly varied between districts. At the district level, specific IgG levels significantly increased with age and decreased with season and with VCM use. The use of insecticide-treated nets, by drastically reducing specific IgG levels, was by far the most efficient VCM regardless of age, season or exposure level to mosquito bites. The use of spray bombs was also associated with a significant reduction of specific IgG levels, whereas the use of mosquito coils or electric fans/air conditioning did not show a significant effect. Human IgG response to gSG6-P1 as biomarker of vector exposure represents a reliable alternative for accurately assessing the effectiveness of malaria VCM in low-malaria-risk areas. This biomarker tool could be especially relevant for malaria control monitoring and surveillance programmes in low-exposure/low-transmission settings.

## Background

Urbanization in Africa is increasing at such a rate that it is estimated that 54% of African residents will live in urban areas by 2030 [1]. Urban development was generally believed to reduce breeding sites of *Anopheles*, and thereby the risk of malaria transmission. However, several factors linked to a rapid and uncontrolled population and/or household growth can have major implications for the disease transmission patterns in cities of sub-Saharan Africa [2], [3]. *Anopheles* vectors can be well adapted to urban settings [4]. Furthermore, even if globally they have low exposure to *Anopheles* bites [5], people living in cities could be at high risk of malarial morbidity and mortality because of their delayed acquisition or lack of protective immunity [3]. The prevalence of malaria in cities is therefore significant and urban malaria has been considered as an emerging public health problem in Africa [6].

Senegal is a country in tropical sub-Saharan Africa with a high rate of urbanization (46.8%) [7]. In 2008, about 23% of its inhabitants lived in Dakar (the largest city), which covers only 0.3% of the country’s area [8]. Malaria risk in the Dakar area is very focal, mainly dependent on the degree and type of urbanization, on the variety of vector control measures (VCMs) in use and on other household factors [9]. To achieve the goal of reducing the burden of malaria towards a pre-elimination stage in the country by 2015, several initiatives have been taken since 2001. The preventive VCMs taken are mainly (i) a widespread use of free insecticide-treated nets (ITNs) by pregnant women and children under 5 years old, (ii) the subsidization of ITNs for other people, and (iii) coverage of 80% of the population by indoor residual sprays (IRS), with particular attention to Dakar suburbs where floods have been recurring since 2004 [8], [10]. In addition, with a better economic situation, health education, and access to healthcare services compared to rural populations, many people in Dakar can easily own and/or use diverse VCMs. This can significantly alter their exposure level to vector bites. Moreover, possible changes in malaria risk patterns in the Dakar area have been linked to the significant increase in building developments between 1996 and 2007 [11]. These findings suggest a current need to accurately assess the risk of malaria in Dakar and generally in low-exposure-level settings.

The evaluation of malaria VCM effectiveness is currently based on entomological methods and, in human populations, on parasitological and clinical assessments. However, these methods are labour-intensive and difficult to sustain on large scales, especially when transmission and exposure levels are low (dry season, high altitude, urban settings or after vector control) [12], [13]. The commonly used entomological method (human-landing catch) to assess the human exposure level to mosquito bites does not give a measure of the individual exposure in a given area. Furthermore, it inevitably increases the hazard of exposure of the participants to mosquito-borne infections and cannot be used with children [14]. Finally, because of the important drop in exposure intensity with the global use of efficient VCMs, current methods evaluating the impact of malaria intervention programs in Africa are less sensitive and less effective, especially in urban areas [15]. A simple, specific and highly sensitive tool is therefore needed for a more accurate surveillance.

The human antibody (Ab) response to mosquito saliva has been described as a new approach capable of predicting the risk of malaria, even at an individual level [16]. Salivary proteins of mosquitoes are injected into the host during the bite and can induce a specific Ab response [17], which represents an indicator of the exposure of the human host to vector bites [16], [18]. Recent studies have described the specific, antigenic and highly conserved (between *Anopheles* species) gSG6-P1 (*An. gambiae* Salivary Gland Protein-6 peptide 1) sequence peptide as a biomarker of *Anopheles* bites in several settings in Senegal [19], [20], [21], including urban areas [22]. The usefulness of such a tool in assessing the real efficacy of ITN use was reported in individuals living in a moderate malaria transmission area of Angola [23]. In addition, a specific IgG response to gSG6-P1 does not seem to build up but wanes rapidly when exposure has failed. This property represents a major strength for its use in the evaluation of human–*Anopheles* contact and of the efficiency of VCM in various epidemiological contexts [23].

To strengthen malaria vector control in low-exposure contexts, the study aimed to validate specific human IgG responses to the gSG6-P1 peptide as an epidemiological indicator evaluating the effectiveness of VCM strategies (single or combined) used by urban populations in Dakar (Senegal). It also highlighted possible determinant factors in the variations of the IgG response to gSG6-P1 salivary peptide.

## Methods

### Study Site

**Figure 1:**
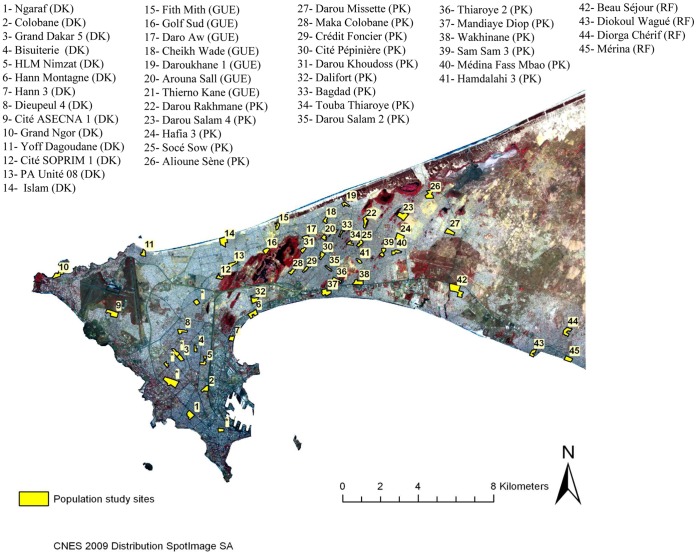
Localization of the studied sites in Dakar. The 50 blood spot-sampling (in yellow) sites are proportionally localized on the map. Enclosed asterisks represent the 5 prosperous residential districts of the Dakar department in which the collected blood samples were not enough for several reasons. The 45 remaining districts in which sufficient blood spot-samples were collected for immunological assays are numbered from 1 to 45 on the map. DK, PK, GUE and RF are, respectively, Dakar, Pikine, Guediawaye and Rufisque, the four departments of Dakar region. The brown base of the map represents the area not inhabited by humans. The darker areas correlate with the presence of vegetation.

Located in the western point of Africa, the Dakar region (14°43′29′′ N, 17°28′24′′ W) had approximately 2,500,000 inhabitants in 2008, amounting to about 21% of the total population of the country (ca. 12,000,000 inhab.), with a high population density (4,459 inhab./km^2^) [7]. This coastal plain area has a mild Sahelian climate with a hot and wet season that lasts from June to November and is characterized by average temperatures ranging from 24 to 30°C. The annual average rainfall in 2008 was 510 mm and peaked in August and September [24]. The study was conducted from the end of the rainy season (October) to the beginning of the dry season (December) in 2008 in 45 sites of downtown Dakar and suburbs (Figure 1). *Plasmodium* species (mainly *P. falciparum*) are transmitted by *Anopheles gambiae s.l.* mosquitoes (mainly *An. arabiensis* and secondarily *An. melas*), and malaria transmission occurs seasonally from July to December, with a peak from September to November [9], [25].

### Study Districts and Populations

The study census districts (CDs, the smallest level in terms of demographic inventory in Senegal) and households were chosen as previously described [22]. Briefly, the first criterion in household selection was the presence of a 2 to 10-year-old child resident. After written agreement from the resident family, investigators presented a questionnaire on the household lifestyle, income and access to healthcare facilities. Concomitantly, the adult woman (generally the child’s mother) who answered the questionnaire was selected for blood sampling. Completed questionnaires included information on: (i) the use of ITNs and other VCMs (mosquito coils, spray bombs, electric fans/air conditioning and incense), (ii) the duration and period of trips to other cities or rural areas during the 3 months preceding blood sampling, (iii) the moment and degree of perception of mosquito bites and (iv) the use of anti-malarial drugs. Complementary information (age, sex and date of sampling) was also reported for each individual selected. Pregnant women, individuals (children and adults) who were sick and/or had taken anti-malarial drugs during the last 15–30 days preceding the nurses’ visit for blood-spot collection on filter paper were not included in the cohort.

In total, 4,658 individuals (2,231 children and 2,427 adults) from 50 CDs of Dakar were sampled for the study. Immunological assays (by ELISA) to evaluate specific IgG responses to gSG6-P1 were carried out on a sub-sample of 2,774 individuals (1,314 children 2–10 years old and 1,460 adults >17 years from 45 CDs) randomly selected within each CD. The number of individuals for whom IgG Ab responses were assessed varied by CD, from 34 to 86.

This study was conducted in accordance with the Edinburgh revision of the Helsinki Declaration, and was approved by the ethics committees of the Ministry of Health and Prevention of Senegal (December 2008). Written informed consent was obtained for all individuals enrolled in the study. For children, this informed consent was signed by one of their parents or their tutor (child guardian).

### Salivary Peptide gSG6-P1

The gSG6-P1-specific *Anopheles* peptide was designed using bioinformatics as previously described [20]. It was synthesized and purified (>95%) by Genepep SA (St-Clément de Rivière, France). All peptide batches were shipped in lyophilized form and then suspended in 0.22-µm ultra-filtered water and frozen in aliquots at −80°C until use for immunological tests (ELISA).

### Evaluation of Human IgG Antibody Levels (ELISA)

Standardized dried blood spots were eluted as previously described [15]. ELISAs were carried out on dried blood-spot eluates to assay IgG response to the gSG6-P1 antigen as described elsewhere [23]. Results were expressed as the ΔOD value: ΔOD = ODx­ODn. ODx and ODn represent the mean of individual optical density (OD) in, respectively, 2 antigen wells and 1 blank well containing no gSG6-P1 antigen. Anti-gSG6-P1 IgG levels were also assayed in non-*Anopheles*-exposed individuals (n = 14- neg; north of France) in order to quantify the non-specific background Ab level and to calculate the cut-off value of the immune response (Co.R = mean (ΔDO_neg_) +3SD = 0.204). An exposed individual was then classified as an immune responder if the ΔOD>0.204.

### Statistical Analysis

With GraphPad Prism5® (San Diego, CA, USA), medians of specific Ab levels to gSG6-P1 between two or more independent groups were compared using the non-parametric Mann–Whitney or Kruskal–Wallis test, respectively. The Fischer exact test was used to compare two proportions. With R software (version 2.14.0) and additional functions from the “nlme” package, the relationship between the anti-gSG6-P1 IgG response (dependent variable) and each explanatory variable was assessed using a univariate linear mixed-effect (LME) model with a random intercept at the “district” level (in this way taking into account possible correlations associated with measurements in each district). We then investigated the combined effects of the explanatory variables on the anti-*Anopheles* gSG6-P1 IgG response peptide using a multivariate LME. All differences were considered significant at the P<0.05 levels.

## Results

### Characteristics of the Study Population

Data are presented for 58.90% (1,314/2,231) of the studied households covering 59.55% (1,314 children and 1,460 adults) of the 4,658 individuals sampled. The age ranged from 2 to 10 years in children and from 17 to 81 years in adults. The median age was 5.0 years in children (Q25% = 3.0 and Q75% = 8.0) and 35.0 years in adults (Q25% = 28.0 and Q75% = 43.0). This was similar between districts, except in adults where it was significantly different only between five pairs of districts (P<0.05). About 51.14% (672/1,314) of children and 89.11% (1,301/1,460) of adults were female.

### Use of Malaria Vector Control Measures

To protect themselves against mosquito bites and malaria disease, 90.01% (2,497/2,774) of the people interviewed declared they frequently used malaria VCM during the 15–30 days preceding the study. ITN, mosquito coils, spray bombs and ventilation (electric fans/air conditioning) were the main VCMs used, at a rate of, respectively, 43.35% (minimum 1.78% in CD1 and maximum 84.61% in CD20), 36.68% (min. 6.15% in CD20 and max. 83.33% in CD13), 9.57% (min. 0.00% in 14 CDs including CD20 and max. 53.45% in CD10) and 7.11% (min. 0.00% and max. 25.76% in CD5) (Table 1). In addition, 4.22% of Dakar residents (min. 0.00% in 15 CDs including CD20 and max. 16.67% in CD11) adopted a combination of these VCMs. Similar patterns in VCM use were also observed when children and adults were separately considered (data not shown). In addition to the inter-district heterogeneity, the use of VCM varied according to season and age. It was significantly higher in 2- to 5-year-old children (47.38%: 308/650) and in adults (43.65%: 628/1,460) compared to 6- to 10-year-old children (37.54%: 232/618) (P = 0.0004 and P = 0.023, respectively) and lower in the second half of October (Oct 2∶38.06%: 110/289) than in the first half of October (Oct 1∶50.00%: 101/202; P = 0.009).

**Table 1:** Proportion of use of vector control measures in the populations of 45 districts of Dakar urban region.

Vector Control Tools	Total Population (n = 2774)	Children (n = 1314)	Adults (n = 1460)
Bed nets	43.35 (±2.76)	43.02 (±2.78)	43.65 (±2.85)
Mosquito coils	36.68 (±2.73)	35.69 (±2.87)	37.58 (±2.77)
Spray bombs	9.57 (±1.71)	8.66 (±1.67)	10.51 (±1.85)
Ventilation	7.11 (±0.94)	6.51 (±0.97)	7.69 (±1.06)
Incense	0.99 (±0.28)	1.11 (±0.32)	1.02 (±0.31)
Others*	4.22 (±0.70)	3.85 (±0.79)	4.97 (±0.79)

The proportion of use in the total population (children and adults), children or adults was calculated for each type of vector control measure listed. The standard error of each proportion is indicated in brackets. “n” represents the effectiveness of individuals in each group. “Others” means simultaneous use of two or more of the listed vector control tools by populations.

### Relationship between the Use of ITN and Other Vector Control Measures

Table 2 shows that 78.99% and 84.74%, respectively, of non-ITN-user children and adults declared they frequently used at least another VCM. However, only 22.91% and 32.01%, respectively, of children and adults used ITN and another VCM concomitantly. Differences were highly significant in the chi-square test (P<0.0001). In addition, the lowest percentages of mosquito coil use (6.15%) and spray bombs (0.00%) were registered in CD20, where the highest percentage of ITN use (84.61%) was observed.

**Table 2:** Relationship between use of bed net and other vector control tools in the study population.

		Children		Adults	
		Bed net use			
		No	Yes	No	Yes
**Other vector control tools use**	No	158 (21.01%)	434 (77.09%)	127 (15.26%)	427 (67.99%)
	Yes	594 (78.99%)	129 (22.91%)	705 (84.74%)	201 (32.01%)
	Total	752 (100.00%)	563 (100.00%)	823 (100.00%)	628 (100.00%)

### Age, ITN Use and Seasonality: Main Factors of Variation of Anti-gSG6-P1 IgG Levels

The combined effects of explanatory variables on the specific IgG response were investigated using a multivariate LME model (Table 3). For all CDs, results indicate that specific IgG responses to gSG6-P1 were significantly lower, for children as well as for adults, in ITN users compared to non-ITN users (P<0.0001 for adults and P = 0.0004 for children). They also significantly decreased with the use of spray bombs alone in adults (P = 0.010) or combined with mosquito coils in children (P = 0.014) *versus* the other non-VCM users. Specific IgG levels also decreased in the second half of November (P = 0.023), first half (P = 0.008) and second half of December 2 (P = 0.008) *vs* the first half of October (the first 15 sampling days). However, no significant specific IgG level variation was observed with sex, trips to other areas, use of mosquito coils alone, ventilation and incense.

**Table 3:** Factors influencing specific IgG response to gSG6-P1 peptide.

Variables	Class	Effective	Estimated parameter	Standard Error	P-value
Children (n = 1314)					
**Intercept**			0.299	0.016	0.0000
**Age** (reference: 2–5 years)	6–10 years	627	**0.055**	**0.010**	**0.0000**
**Bed-nets** (reference: not used nets)	used net	546	**−0.044**	**0.012**	**0.0004**
**other vector control methods** (reference: not used other VCM)	mosquito coils	464	0.007	0.013	0.5950
	spray bombs	124	**−**0.002	0.019	0.9351
	mosquito coils+spray bombs	28	**−0.086**	**0.035**	**0.0142**
	Ventilation	82	0.000	0.022	0.9978
	mosquito coils+ventilation	14	0.023	0.050	0.6396
	spray bombs+ventilation	12	**−**0.080	0.053	0.1371
	incense	12	**−**0.026	0.052	0.6087
Adults (n = 1460)					
**Intercept**			0.300	0.013	0.0000
**Period of Sampling** (reference: 01–15 Oct. 2008)	16–30 Oct.	290	**−**0.034	0.030	0.2633
	01–15 Nov.	275	**−**0.055	0.038	0.1510
	16–30 Nov.	331	**−0.080**	**0.035**	**0.0230**
	01–15 Dec.	168	**−0.097**	**0.037**	**0.0087**
	16–30 Dec.	186	**−0.096**	**0.036**	**0.0079**
**Bed-nets** (reference: not used nets)	used nets	628	**−0.064**	**0.013**	**0.0000**
**other vector control methods** (reference: not used other VCM)	mosquito coils	541	**−**0.011	0.014	0.4170
	spray bombs	158	**−0.050**	**0.019**	**0.0104**
	mosquito coils+spray bombs	40	**−**0.014	0.033	0.6755
	Ventilation	112	**−**0.005	0.021	0.8016
	mosquito coils+ventilation	20	**−**0.082	0.045	0.0672
	spray bombs+ventilation	14	**−**0.036	0.053	0.4983
	incense	15	0.027	0.051	0.5924

Intercept = when the values of all independent variables are zero (e.g. the value of median IgG response in someone with no risk factors). The estimated coefficient and the degree of significance (p-value) are indicated. A positive coefficient means that the explanatory variable increases the probability of IgG response to gSG6-P1, while a negative coefficient means that the variable decreases the probability of IgG response to gSG6-P1.

Age had a significant impact on the anti-gSG6-P1 IgG response. Indeed, the specific IgG level was higher in children aged 6–10 years *vs* 2–5 years (P<0.0001) and in adults *vs* all children (P<0.0001). Moreover, in the majority of CDs, the median of anti-gSG6-P1 levels was higher in adults than in children. However, this difference was only significant in CD n°12 (P = 0.01).

### Specific IgG to gSG6-P1 Peptide for Assessing Effectiveness of ITN Use in Urban Areas

**Figure 2:**
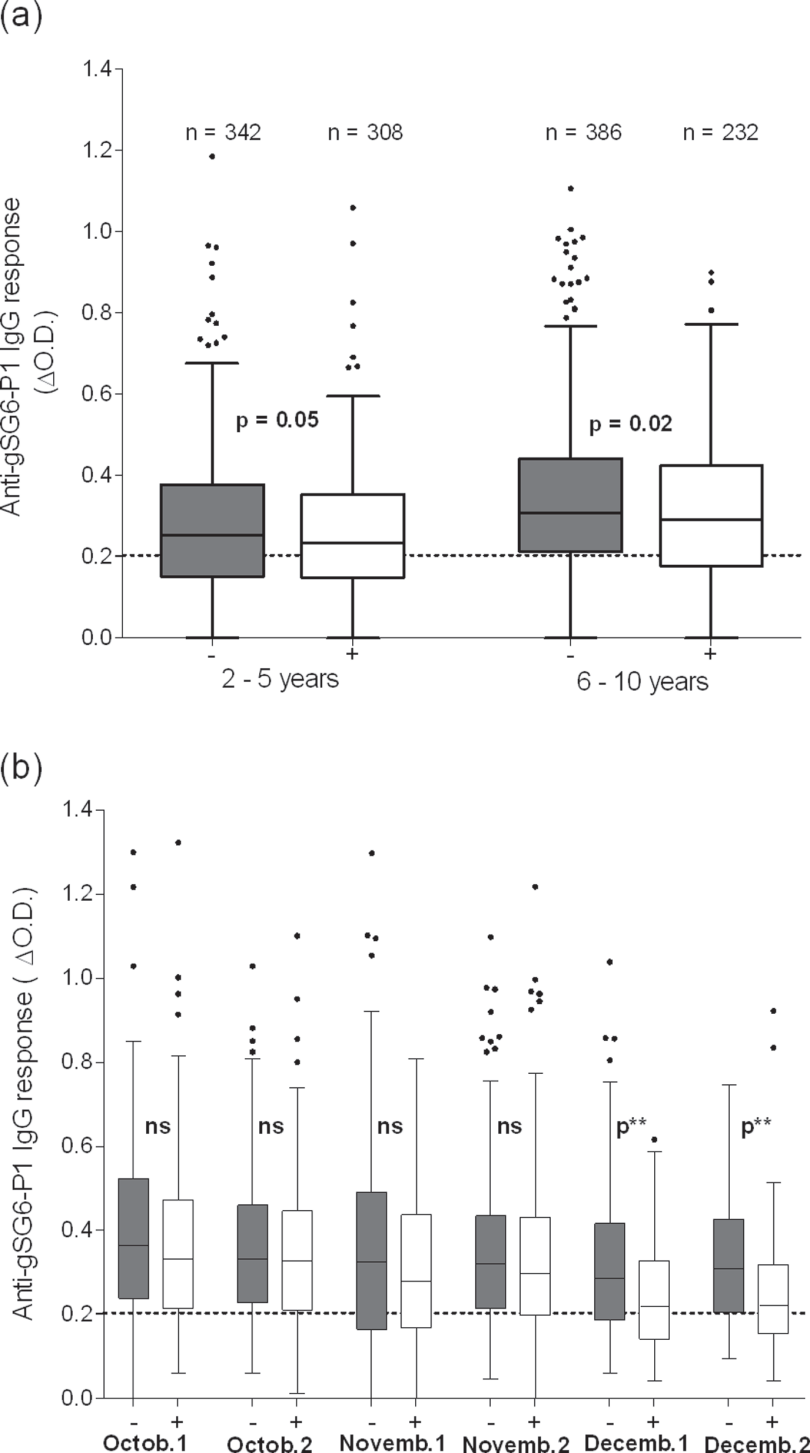
IgG responses to gSG6-P1 according to the use of ITN and age (in children) and sampling period (in adults). Specific IgG responses are shown for ITN (white boxes) and non-ITN (grey boxes) users according to age in children (Fig. 2a) and period of sampling in adults (Fig. 2b). Boxes indicate the middle 50% of the data; horizontal lines in the boxes indicate medians of the individual data; lengths of boxes correspond to the inter-quartile ranges. The horizontal black dotted line represents the cut-off of IgG responder and “n” the effectiveness of each individual group. Statistical significant differences of specific IgG between bed net and non-bed net users are indicated. October 1 and 2, November 1 and 2 and December 1 and 2 represent sampling periods between, respectively, 01^st^ –15^th^ and 16^th^ –31^st^ October, 01^st^ –15^th^ and 16^th^ –30^st^ November and 01^st^ –15^th^ and 16^th^ –31^st^ December 2008.

Regardless of the results on determinant factors of variation, anti-gSG6-P1 IgG levels are represented according to ITN use for children aged 2–10 years and 6–10 years (Fig. 2a) and for the different sampling periods in adults (Fig. 2b). Results indicate that anti-gSG6-P1 IgG levels were significantly lower in ITN users regardless of age: 2–5 years old (P = 0.05) and for 6–10 years old (P = 0.02). In adults, they were also significantly lower in ITN users regardless of the month of sampling. However, this difference was only significant in December (P<0.001 for the first and second half of December). Results also showed that the impact of ITN use seemed to be more significant in children aged 6–10 years than those aged 2–5 years (see Fig. 2a) and in adults (P<0.0001) compared to all children (P = 0.0009).

### Specific IgG to gSG6-P1 Peptide for Estimating the Perception Level of *Anopheles* Bites

**Figure 3:**
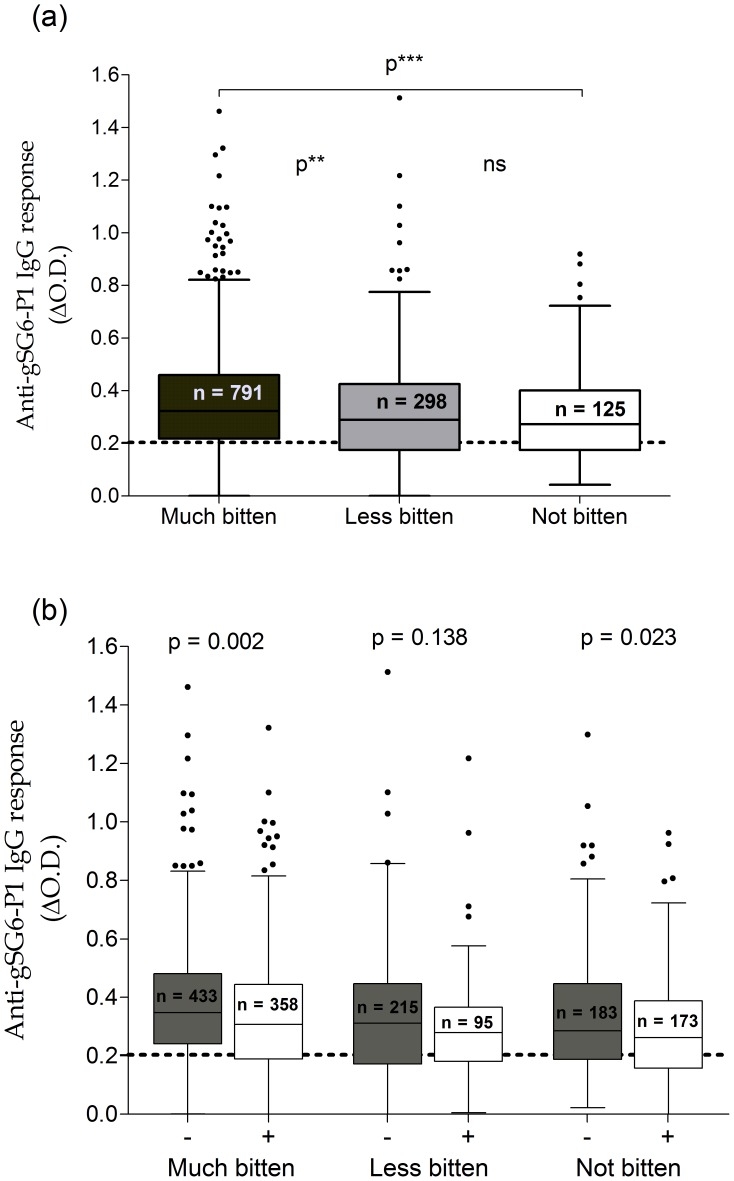
IgG responses to gSG6-P1 according to adult perception of mosquito bites. Anti-gSG6-P1 IgG responses represented according to the degree of perception of mosquito bites (Fig. 3a) and taking into account the use of bed nets (Fig. 3b). Boxes indicate the middle 50% of the data; horizontal lines in the boxes indicate medians of the individual data; lengths of boxes correspond to the inter-quartile ranges. In Fig. 3B, bed net and non-bed net users are represented white and grey boxes, respectively. The horizontal black dotted line represents the cut-off of IgG responder. Statistical significant differences of specific IgG between bed net and non-bed net users are indicated.

When asked: *“Have you been bitten by mosquitoes? If yes, when?”*, 74.50% (1,089/1,460) of adults declared they were bitten by mosquitoes at night (from 7∶00 pm to dawn). Among them, 72.65% (791/1,089) and 27.36% (298/1,089) were bitten to a high and a low degree, respectively. Anti-gSG6-P1 IgG levels were compared between the high-degree, low-degree and non-bitten groups (Fig. 3a). The median of specific IgG levels was higher in the group with a high-degree perception of mosquito bites compared to the low-degree and non-bitten groups (P<0.01 and P<0.001, respectively). However, no significant difference was observed between the low-degree and non-bitten groups (P>0.05).

The proportion of ITN use was 45.51% (360/791), 31.54% (94/298) and 48.59% (173/356) in high-degree, low-degree and non-bitten adults, respectively (data not shown). Differences were only significant when comparing the low with the high-degree and non-bitten groups (P<0.0001). Specific IgG levels were also represented according to ITN use (Fig. 3b). Results indicated that IgG levels were lower in ITN users, regardless of the group of perception. However, this difference was only significant in the high-degree and non-bitten perception groups (P = 0.023 and 0.002, respectively).

## Discussion

The present study focused on the application of the specific *Anopheles* gSG6-P1 salivary peptide as a biomarker in the evaluation of the effectiveness of a range of malaria VCMs in urban low-endemic malaria settings.

The use of VCMs in the Dakar region was assessed by a socio-epidemiological questionnaire. To protect themselves against the bites of adult malaria vectors, the urban dwellers of Dakar, who were interviewed, used ITNs (alone or combined), mosquito coils, spray bombs, ventilation and/or incense. This variety suggests great socio-economic and cultural discrepancies between household, as has been described in large cities of the Ivory Coast [26] and Tanzania [27]. ITNs were the first-choice preventive VCM in Dakar. This could be partly linked to the national malaria control policy in Senegal which mainly promotes their use [28]. However, assessing ITN use by socio-epidemiological surveys can lead to overestimation [29]. Moreover, a lower rate of ITN use (16.6%) was reported by the National Malaria Control Program (NMCP) of Senegal during the transmission season in 2008, covering the study period, even if the sampled populations, study period and methods used were different [28]. Reasons of non-ITN use were not addressed in the present study, whereas in the NMCP surveys the absence of mosquitoes (46.4%) and temperature (16.3%) were the most frequently cited reason [28]. The significant use of mosquito coils can probably be attributed to their low price (about 0.15$ US/unity) and high availability in markets and intra-district shops. Spray bombs were less frequently used, certainly because of their recent adoption and their higher cost in the majority of sub-Saharan Africa cities [30]. The combined use of VCMs listed above was not frequent. In addition, the use of other VCMs was negatively associated with ITN use. This confirms observations in Tanzania, and means that people use other VCMs when ITNs are inaccessible for various reasons [27].

The anti-gSG6-P1 IgG response has been described as an alternative tool for a direct and accurate measure of the human exposure level to *Anopheles* bites in several settings [19], [23]. In this study, such an approach was applied to evaluate the effectiveness of different malaria VCMs in an urban context. The high intra−/inter-district heterogeneity in the specific IgG Ab levels confirms recent findings highlighting great differences in human–*Anopheles* contact and the risk of malaria in the Dakar area [22]. This could be explained by differences in the use of VCMs [27], the height and type of households, individual age and sex, sleeping behaviour [23], seasonality [22] or movements of the populations. The use of ITN and spray bombs, age and seasonality only showed significant effects on child and/or adult specific IgG responses. The increase seen with age has been previously described [23] and seems to be consistent with the gradually acquired malaria immunity [31] and the development of individual factors and behaviours enhancing the probability of human–vector contact [27], [32]. The effect of sex was not significant, confirming results obtained in Angola [23], even if the specific IgG level appeared globally higher in females, as previously described [33]. Travelling to other areas can have significance in malaria risk transmission in a given region [34]. In this study, few individuals declared having travelled to rural or other urban areas within/out the country during the 3 months before the study. The majority did not have a clear idea of the period and/or duration of their trip, perhaps explaining the absence of an effect of this parameter. Anti-gSG6-P1 IgG levels in adults waned from the beginning (October) to the end (December) of the study. This is certainly due to an important drop in human exposure level to *An. gambiae s. l.* bites from the end of the rainfalls (October) to the beginning of the dry season (December) [9], [25]. Regardless of the effects of VCM use, ITN – by reducing drastically the human–*Anopheles* contact – was the main factor for the drop in anti-gSG6-P1 IgG levels in children as well as in adults, whatever the individual age, period of sampling or the perception level of mosquito bites. Spray bombs were secondarily associated with a decrease of specific IgG levels, certainly due to their power and fast knock-down action. However, their effect can be limited by the non-persistence of used products and some socio-economic considerations [30]. The non-effect of mosquito coil use is surprising, since coils have been well-adopted by residents; however, it can be explained by their powerful deterrent effect, which tends to push *Anopheles* vectors outside where they can remain active [30]. Taken together, these results suggest that the assessment of human Ab responses to *Anopheles* gSG6-P1 salivary peptide can provide a reliable evaluation of the effectiveness of malaria vector control in urban settings of Dakar, regardless of the age, sex, level of exposure to bites or period of malaria transmission.

The gradual age-dependent impact of ITN on specific IgG levels seemed to reflect a probable increase of exposure level rather than the rate of ITN use. In December, a period of very low presence or absence of *Anopheles* in the area, the lower rate of ITN use dramatically reduced human–*Anopheles* contact and specific IgG levels in contrast to October and November when vector densities were relatively higher. Therefore, the protection offered by ITN use could be insufficient in October–November, as shown by the values of median anti-gSG6-P1 IgG levels in ITN users, which were over the cut-off of the immune responder line. Changes in *An. arabiensis* behaviour, the major malaria vector in the area, can also explain this lack of protection. It can bite outside rooms/habitations with a maximal activity around 10.00 pm, when people are not in bed and ITNs are not hanged [27]. *An. melas*, *An. pharoensis* and *An. ziemmani* have also been described locally in Dakar and *An. melas* secondarily associated with malaria transmission [25]. It can be hypothesized that the impact of VCMs on these anthropophagic species was also evaluated by using this highly conserved *Anopheles* salivary gSG6-P1 peptide [20], [35]. For an effective prevention of adult *Anopheles* bites, it is suggested to combine ITN use with a complementary VCM: door and window screenings, mosquito coils and sprays [26], anti-larval [36] and environment control [37]. Unfortunately, the adoption of other VCMs in Dakar is highly dependent on the use of ITNs. Therefore, populations in endemic cities of Africa must be sensitized to combine several VCMs for effective prevention of malaria.

The results from the analysis of the epidemiological questionnaires on the perception of mosquito bites at night are surprisingly interesting. The median of IgG levels to gSG6-P1 was significantly higher in the highly bitten group and similar between the low and non-bitten groups. This observation suggests that the assessment of the level of perception of mosquito bites by a simple questionnaire can be a credible alternative where entomological data are unavailable or limited. However, this parameter can be particularly limited because it cannot be applied to children and because of an eventual lack of objectivity and reliability in the questionnaire’s answers. Nonetheless, the statistical significance of results in this study support the assertion that assessment of IgG response to gSG6-P1 peptide could be a useful indicator for evaluating the level of human perception of *Anopheles* bites. This serological biomarker therefore appears to be a credible alternative to current socio-epidemiological survey methods assessing the effectiveness of malaria VCM.

### Conclusions

This study highlights the relevance of the Ab response to the specific *Anopheles* gSG6-P1 salivary peptide in reliably evaluating the effectiveness of malaria VCMs in urban settings, at both population and individual levels. ITN, by reducing more significantly the probability of human–*Anopheles* vector contact, was the most effective malaria VCM. However, it must be combined with other anti-vector method for sufficient prevention. This approach can be a credible alternative to standard entomological survey methods particularly in low-exposure areas, and especially in urban settings. It must be applied in epidemiological malaria studies by taking into account several parameters such as individual age, season of transmission of the concerned disease and use of anti-vector devices. This serological method could also allow for pertinent monitoring and surveillance of anti-vector strategies developed by the National Vector-borne Disease Control Programmes in urban sub-Saharan Africa.
